# Supplementary figures and images for: Production location of the gelling agent Phytagel has a significant impact on Arabidopsis thaliana seedling phenotypic analysis
Source: PLoS One. 2020 May 14;15(5):e0228515. doi: 10.1371/journal.pone.0228515 (PMC7224531; doi:10.1371/journal.pone.0228515)

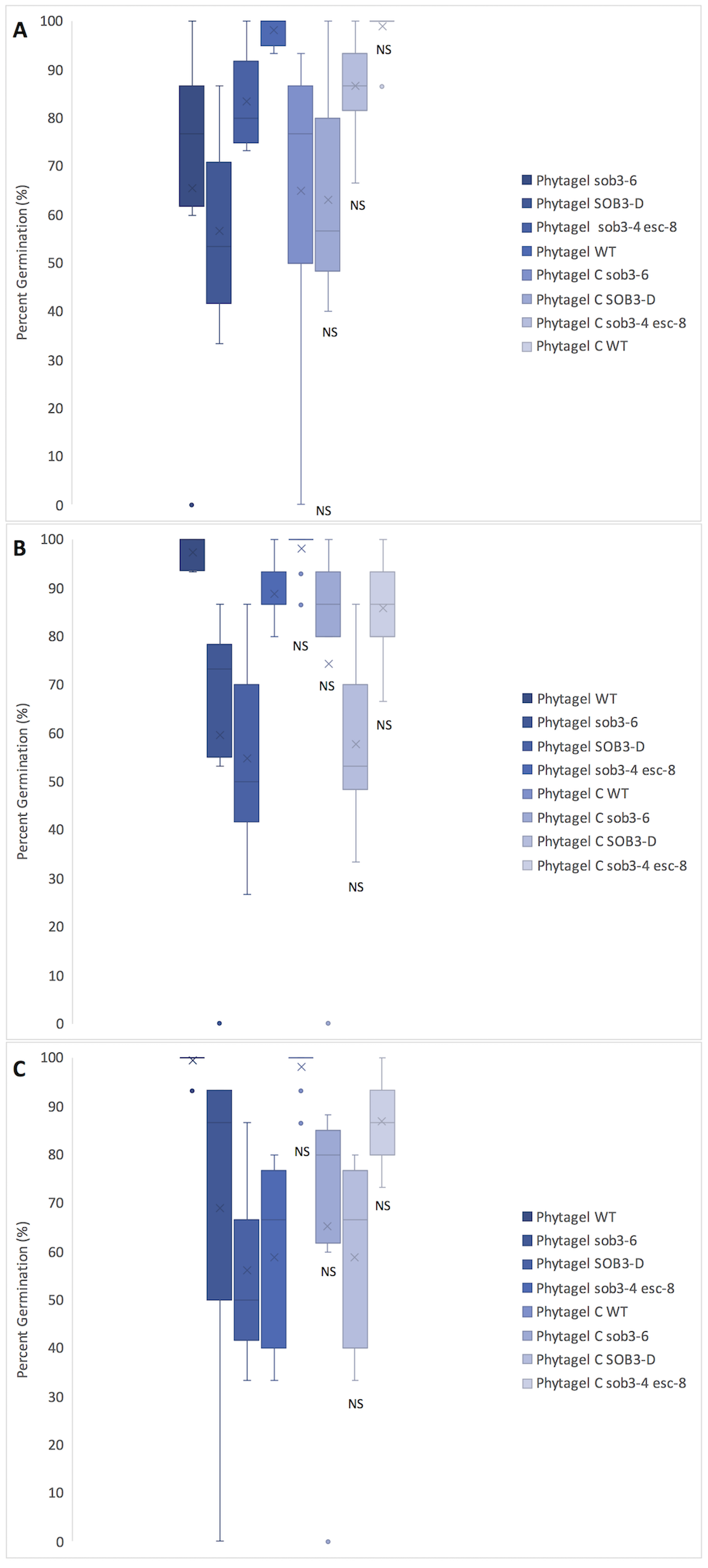

Supplement: S1 Fig — A) Germination rates of WT, sob3-6, SOB3-D, and sob3-4 esc-8 at 10 μmol m-2s-1 on 1% Phytagel and 1% Phytagel C plates. B) Germination rates of WT, sob3-6, SOB3-D, and sob3-4 esc-8 at 60 μmol m-2s-1 on 1% Phytagel and Phytagel C plates. C) Germination rates of WT, sob3-6, SOB3-D, and sob3-4 esc-8 at 100 μmol m-2s-1 on 1% Phytagel and Phytagel C plates. In a Welch’s t test (unpaired two-tailed t test with unequal variance) compared with the wild type, P > 0.05 = Not Significant (NS). (TIFF) [file pone.0228515.s001.tiff]

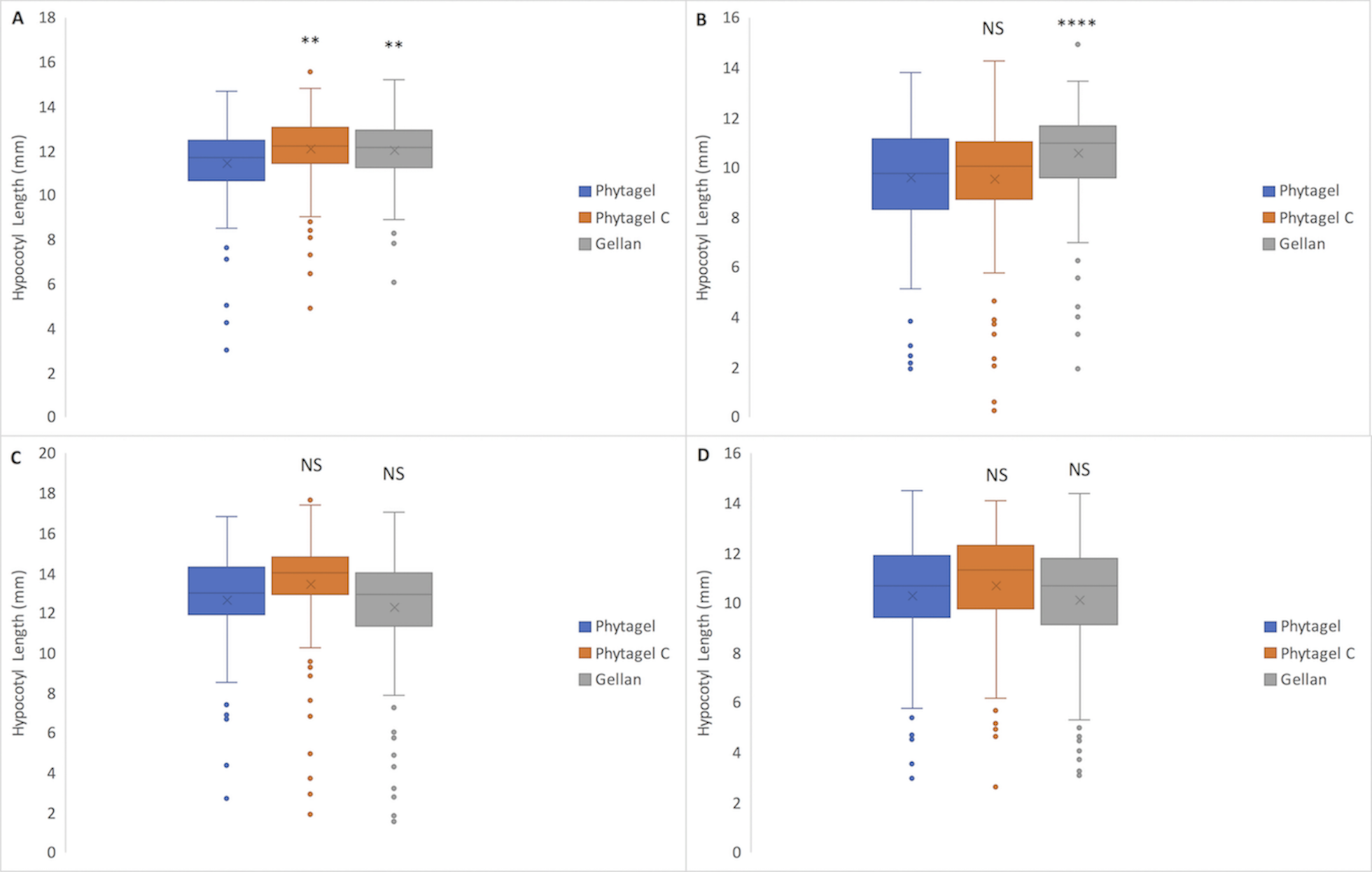

Supplement: S2 Fig — A) Hypocotyl lengths of WT seedlings on different growth media plates. In a Welch’s t test (unpaired two-tailed t test with unequal variance) compared Phytagel, P ≤ 0.01 = **, P > 0.05 Not Significant (NS). B) Hypocotyl lengths of sob3-4 esc-8 seedlings on different growth media plates. In a Welch’s t test (unpaired two-tailed t test with unequal variance) compared Phytagel, P ≤ 0.0001 = ****, P > 0.05 = Not Significant (NS). C) Hypocotyl lengths of sob3-6 seedlings on different growth media plates. In a Welch’s t test (unpaired two-tailed t test with unequal variance) compared Phytagel, P > 0.05 = Not Significant (NS). D) Hypocotyl lengths of SOB3-D seedlings on different growth media plates. In a Welch’s t test (unpaired two-tailed t test with unequal variance) compared Phytagel, P > 0.05 = Not Significant (NS). (TIFF) [file pone.0228515.s002.tiff]
